# Investigating the evolution and development of biological complexity under the framework of epigenetics

**DOI:** 10.1111/ede.12301

**Published:** 2019-07-03

**Authors:** Kevin K. Duclos, Jesse L. Hendrikse, Heather A. Jamniczky

**Affiliations:** ^1^ Department of Cell Biology and Anatomy The University of Calgary Calgary Alberta Canada; ^2^ Department of Community Health Sciences The University of Calgary Calgary Alberta Canada

**Keywords:** biological complexity, development, epigenetics, evolution, hybridization

## Abstract

Biological complexity is a key component of evolvability, yet its study has been hampered by a focus on evolutionary trends of complexification and inconsistent definitions. Here, we demonstrate the utility of bringing complexity into the framework of epigenetics to better investigate its utility as a concept in evolutionary biology. We first analyze the existing metrics of complexity and explore the link between complexity and adaptation. Although recently developed metrics allow for a unified framework, they omit developmental mechanisms. We argue that a better approach to the empirical study of complexity and its evolution includes developmental mechanisms. We then consider epigenetic mechanisms and their role in shaping developmental and evolutionary trajectories, as well as the development and organization of complexity. We argue that epigenetics itself could have emerged from complexity because of a need to self‐regulate. Finally, we explore hybridization complexes and hybrid organisms as potential models for studying the association between epigenetics and complexity. Our goal is not to explain trends in biological complexity but to help develop and elucidate novel questions in the investigation of biological complexity and its evolution.

## RESEARCH HIGHLIGHTS

This manuscript argues that biological complexity is better understood under the framework of epigenetics and that the epigenetic interactions emerge from the self‐regulation of complex systems. Hybrids are offered as models to study these properties.

## Graphical abstract

Biological complexity is the result of the hierarchical organisation of nested levels of cells, tissues and higher order part types interacting together.

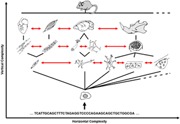

## 1 INTRODUCTION

Complexity, commonly defined as the number of parts composing a system, is a pivotal concept in evolutionary biology (McShea & Brandon, 2010; Wagner & Altenberg, 1996; Wagner & Zhang, 2011). Complexity and how it has increased throughout evolution have been central to evolutionary thinking and studies since the publication of “*On the origin of species by means of natural selection*” by Charles Darwin (Darwin, 1859; McShea, 1991, 1996; Valentine, Collins, & Meyer, 1994). More recently, the idea that more complex systems are more evolvable has led to renewed attention to biological complexity and its evolutionary bases (McShea & Brandon, 2010; Wagner & Altenberg, 1996; Wagner & Zhang, 2011). Further, complexity seems linked to how many different tasks organisms can perform and how well they cope with new environmental challenges (Adami, 2002; Adami, Ofria, & Collier, 2000; Carroll, 2001; Waddington, 1969). Evolutionary biologists have argued that increasing complexity is an observable trend in evolution (Adami et al., 2000; McShea, 2005; Orr, 2000; Valentine et al., 1994) and some maintain that increasing complexity is the background tendency (i.e., the tendency for systems upon which no directional evolutionary forces act) for evolving systems (McShea, 2005, 2017). Conversely, complexity can be costly to produce and maintain (Csete & Doyle, 2002; Orr, 2000), and, under natural conditions, there may be an upper limit as to how complex organisms can become (Heim et al., 2017). Investigating how body plan complexification and structural changes affect evolvability and ecological variability is of critical interest. This is especially true in the context of rapid environmental changes, such as those seen in the Holocene. Despite its importance, the study of complexity and its evolution have been hindered historically by the variety of definitions that have been used for complexity and by authors treating increases in complexity as de facto favorable outcomes in evolutionary trajectories.

Modern definitions of complexity which readdress the link between complexity and selection have been proposed, raising new questions regarding the adaptive value of complexity and how complexity is assembled (McShea & Brandon, 2010; Barrett et al., 2012). Though these recent accounts of complexity offer operational definitions, aspects of development such as cell differentiation, gene regulation, and regulatory mechanisms, in general, continue to be neglected. Moreover, despite authors defending the idea that increases in complexity are beneficial (Wagner & Altenberg, 1996), evolutionary trends toward reduction in complexity are identifiable (Sidor, 2001; Wolf & Koonin, 2013). Complexity thus appears to be highly dynamic both in development and in evolution.

The adaptive value of complexity may be better assessed by including complexity in an evolutionary‐developmental biology framework with a focus on the role of developmental mechanisms in producing or limiting phenotypic variation. Despite the acknowledged role of epigenetic mechanisms in mediating developmental plasticity, gene regulation, and posttranscriptional modifications, the degree to which epigenetic mechanisms participate in the organization of complexity has received very little attention.

We argue that epigenetics and complexity are inextricably linked concepts and that epigenetic mechanisms and their origination should be included in our empirical assessments of biological complexity and its evolution. To illustrate the utility of using epigenetics as a framework to study the evolution and development of complexity, we show how hybrid lineages provide a powerful tool for the study of epigenetic mechanisms in cases of rapid complexity changes or stasis and provide a unique insight into the relationship between these processes in evolution. The structure of this essay is as follows: first, older and more modern definitions of complexity and the adaptive role of complexity are reviewed, and the relationship between development and complexity and the need for a regulatory architecture for complexity are outlined; second, definitions of epigenetics, both as originally conceived by Waddington and other, expanded definitions, are reviewed and evaluated, the roles that epigenetic mechanisms play in shaping developmental and evolutionary trajectories are explored, and the idea that epigenetics emerged from a need for complexity to self‐regulate is subsequently defended; finally, we suggest that hybridization complexes and hybrid lineages could be used as natural models to study how epigenetic mechanisms affect the evolution of biological complexity, given that hybridization is known to be a process by which evolutionary novelties can arise quickly and by which new evolutionary lineages are formed, hybridization may provide examples of rapid (i.e., across a single or few generations) changes in complexity (Barton, 2001; Seehausen, 2004; Soltis, 2013).

### 1.1 Development and complexity

#### 1.1.1 Defining complexity

Complexity is a recurring idea in evolutionary biology (Carroll, 2001; McShea & Brandon, 2010; Wagner & Altenberg, 1996), sometimes being the object of study and sometimes being mentioned without reference to the actual biological meaning and role of complexity (Hansen, 2006; Renaud, Alibert, & Auffray, 2012; Brennan & Keverne, 2015). However, the varied uses of the term and its disparate definitions have resulted in inconsistencies in asking and answering questions about the evolution and adaptive role of complexity (McShea & Brandon, 2010). Early on, complexity was defined as the size of the minimum descriptor of a system (Hinegardner & Engelberg, 1983; Monod, 1970; H. Simon, 1962; Valentine, 2000). In the intervening years, “complexity” has been used to refer to the number of genes in a given genome (a definition that itself is very sensitive to the definition of what a gene is; A. P. Bird, 1995; Brem & Kruglyak, 2005; Gregory, 2002; van Regenmortel, 2004), the number of morphological traits composing an individual (Akam, 1995; Carroll, 2001; Valentine, 2000; Valentine et al., 1994), the number of different cell types found in an organism (Carroll, 2001), the number of developmental processes and pathways involved in the development of an organism (McShea, 1996), along with various combinations of these attributes of organisms. Despite this apparent dissonance among authors, all definitions shared the idea that complexity should be defined as “the number of parts” (i.e., genes, cells, tissues, traits, and pathways) composing an organism. However, though this common theme underlies the various definitions of complexity, empirical studies have mostly tended to restrict their approach to specific sets of parts at particular levels of biological inquiry and have not investigated relationships between levels. The insulated interests of authors in the empirical study of biological complexity have possibly inhibited our understanding of the evolution of organismal form and the relationship between the different levels of biological organization.

**Figure 1:**
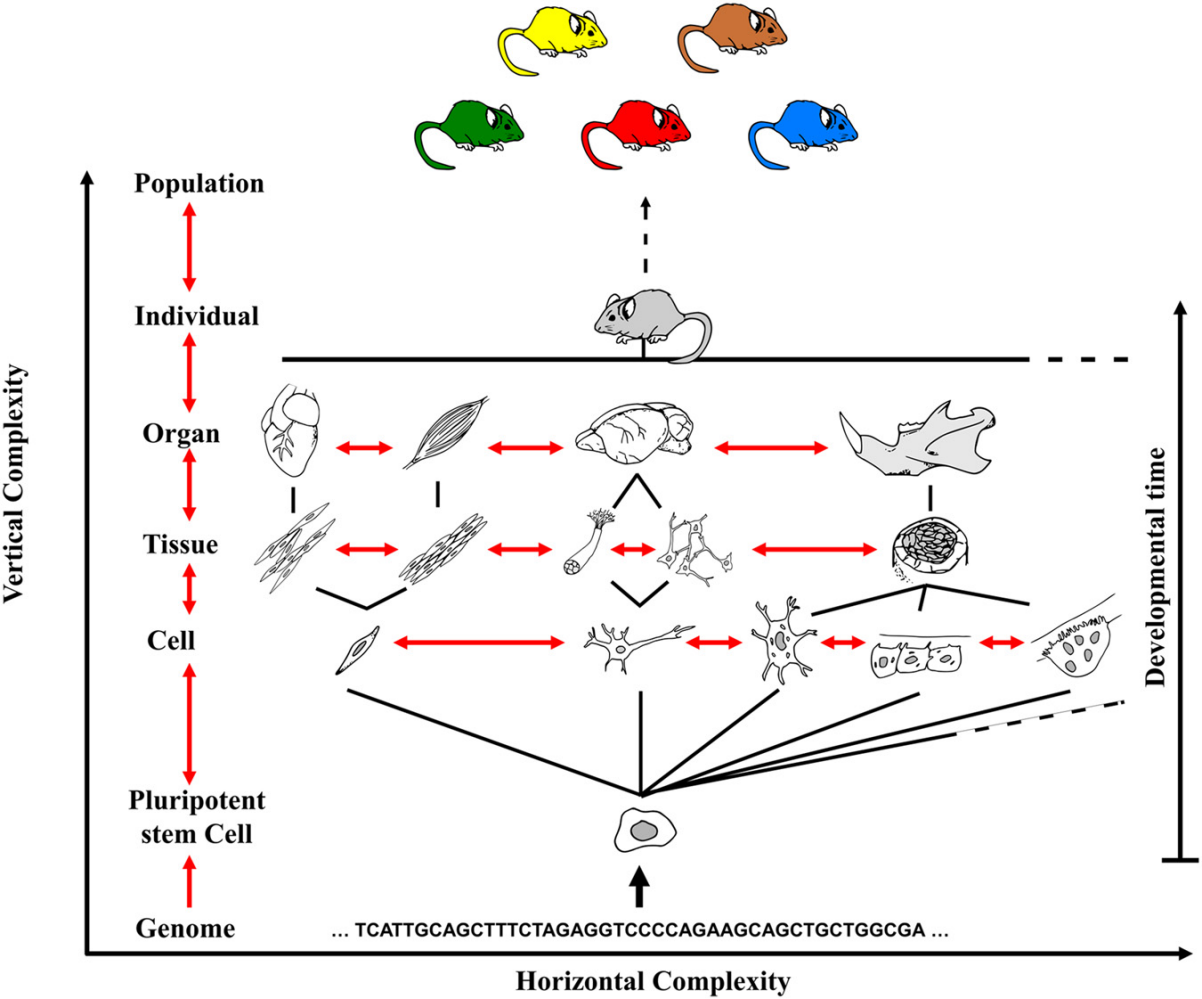
The hierarchical quality of complexity during development. Organismal complexity and the interactions across nested levels of complexity. Two measures of complexity can be obtained. The first measure, horizontal complexity is assessed at a single level of biological organization (i.e., number of different cell types, or number of different organs). The second measure, vertical complexity refers to the numbers of hierarchically nested steps between the lowest rank of biological organization, of the system studied, and the highest. Red arrows show interactions between parts at a given level and between different nested levels. The diagrams represent animal cells, tissues, and organs by convenience; however, the same ideas are applicable to non‐animal organisms [Color figure can be viewed at wileyonlinelibrary.com]

Efforts to create a universal theory of complexity date back to the 1960s and the seminal paper “*The architecture of complexity*” by H. Simon (1962). This paper led to a renewed focus on complexity in systems biology and computational science with many parallels being drawn between engineered and natural systems. Recently, however, authors in the fields of evolutionary biology have worked towards delivering operational definitions of complexity more specifically targeted at biological systems, and which are applicable to all levels of biological organization and allow for the relationships between these levels to be addressed (McShea, 1996, 2017; McShea & Brandon, 2010; McShea & Changizi, 2003). Operational definitions for complexity have been reformulated by McShea (2017), where complexity is split into two components: horizontal complexity and vertical (or hierarchical) complexity. Horizontal complexity is defined as the number of different parts composing a system (Figure 1). These parts can be molecules, proteins, cells, tissues, organs, functional structures, or any unit of description that is quantifiable or qualifiable in any way. This definition retains the logic of the definitions previously discussed but each level of horizontal complexity is inscribed within vertical complexity, that is, the number of hierarchically nested levels of biological complexity (Figure 1). Vertical complexity thus describes how cells assemble into tissues which assemble into organs which eventually form organisms. These definitions of complexity are very broad, potentially referring to any and all types of biological objects from genes and molecules to whole communities, but they allow for all research on complexity to be regrouped under a single framework capturing all types of biological objects and their structure (McShea, 2017; McShea & Brandon, 2010). These definitions of complexity are the ones we adopt for the remainder of this paper and our usage of the term “complexity” hereafter refers to overall complexity as determined by both horizontal and vertical complexity.

#### 1.1.2 Complexity and adaptation

The definitions reiterated in McShea and Brandon (2010) and McShea (2017) are central to the concept of the Zero‐Force Evolutionary Law (ZFEL; Korb & Dorin, 2011). In short, this law stipulates that objects upon which no directional evolutionary forces act, and free of developmental or functional constraints, will tend to complexify and diversify as a result of random change mechanisms, such as drift (McShea, 2016; McShea & Brandon, 2010). The natural background tendency for organisms, in the absence of constraints and selection, would thus be to become more complex and more diverse over evolutionary and developmental time. The ZFEL offers a null model to investigate the evolution of complexity, but, as acknowledged by its authors (McShea & Brandon, 2010), organisms are seldom, if ever, free of constraints and selective pressures, and changes in the magnitude of complexity do not themselves offer advantages (McShea & Brandon, 2010; O'Malley, Wideman, & Ruiz‐Trillo, 2016). Rather, these changes might be contextually selected or counter‐selected.

Complexity may be useful under certain circumstances. Many authors have maintained that increasing complexity could provide more substrate for selection and therefore could promote evolvability (Adami et al., 2000; Csete & Doyle, 2002; Lynch, 2007; McShea, 2017). Waddington (1969) argued that more complex organisms could access novel, more complex, niches, characterized by new interspecific interactions and habitats (Adami, 2002; Carroll, 2001; McShea, 1991). This idea has been supported by studies of simulated populations (Korb & Dorin, 2011; LaBar & Adami, 2016). Heim et al. (2017) report that increased vertical complexity may have led to physiological performance increases in multiple lineages and may have facilitated access to previously restricted habitats. Complexity has also been linked to phenotypic novelty and specialization of structures. For instance, the Weberian ossicles in ostariophysan fish represent an increase in complexity of vertebral and associated neural elements that have become functionally coopted into a specialized hearing mechanism (N. C. Bird & Hernandez, 2009; Grande & de Pinna, 2004; Grande & Young, 2004). Finally, some authors have asserted that complexity could improve the functional robustness of biological systems by exploiting redundancies and many‐to‐one mapping of traits to function (Alfaro, Bolnick, & Wainwright, 2005; Csete & Doyle, 2002). Changes in complexity thus appear to promote evolvability by enabling access to new niches, developing of new functional traits and by making organisms more robust to both environmental and phenotypic changes (J. M. Carlson & Doyle, 2002; Csete & Doyle, 2002; Lenski, Ofria, Collier, & Adami, 1999).

Conversely, costs and potential limits to complexity have been identified (Csete & Doyle, 2002; Cheverud, 2008; Heim et al., 2017; McShea, 1996; Orr, 2000). Decreases in complexity have been identified as a likely consequence of rapid and extreme changes in environmental conditions (Adami, 2002). These decreases occur when overlying complex systems become maladapted to their environment (Adami, 2002). Moreover, many authors anticipated that universal pleiotropy (i.e., the idea that all genes affect all traits, or in its modern version, that most genes affect many traits; Paaby & Rockman, 2013; Pavlicev & Wagner, 2012) would introduce a cost to complexity in the form of reduced adaptive speed (Orr, 2000; Wagner et al., 2008; Wang, Liao, & Zhang, 2010; Welch & Waxman, 2003). Despite recent work indicating pleiotropy to be less universal than originally thought (Hill & Zhang, 2012; Wagner & Zhang, 2011), the propensity of genes to affect multiple traits remains a factor affecting the evolution of complexity (Wang et al., 2010; Wagner et al., 2008). Other limits to complexity might also be at play. Some may be intrinsic, such as the size limits of genomes and functional limits to growth in metazoans (Adami, 2002; Gregory, 2002; Heim et al., 2017; Payne et al., 2011). Others might be due to selective pressures or developmental constraints that can both limit and bias changes in complexity within systems (Heim et al., 2017; McShea & Brandon, 2010). Ultimately, an increasing number and variety of parts in a complex system may involve an increasing number of physical and developmental interactions among these parts, possibly inhibiting evolvability rather than promoting it (Kitano, 2002). In addition, while complex systems may be more robust to some perturbations because of redundancy and compensatory mechanisms, they may paradoxically be more susceptible to these perturbations and potentially deleterious effects because of increase interdependencies and interactions between parts (Albert, Jeong, & Barabási, 2000; Kitano, 2002).

#### 1.1.3 Development of complex organisms

The applications of early definitions of complexity, and to some extent the modern definitions, in research often overlooked aspects of biological organization that are central to our understanding of evolution. For example, original approaches to the study of genetic complexity were very reductive. The idea of biological complexity as being a function of the number of genes stems from a gene‐deterministic point of views assuming a one‐to‐one product (A. P. Bird, 1995; A. P. Bird & Tweedie, 1995; Carroll, 2001). Yet, noncoding sequences can have as much importance as coding sequences in the development of a trait (Costa, 2008; Hornstein & Shomron, 2006; Taft, Pheasant, & Mattick, 2007), and data on gene duplication, pleiotropy, epistasis, and regulatory networks has shifted the understanding of genetic architecture to include noncoding and regulatory DNA (Carroll, 2001; Erwin & Davidson, 2009; Woolfe et al., 2004; Wray, 2007).

As suggested by Bird, gene numbers cannot increase consistently within organisms without regulatory mechanisms to silence some genes and control context‐specific expression to limit transcriptional noise (A. P. Bird, 1995; A. P. Bird & Tweedie, 1995). The evolution of Eukaryotes depends strongly on regulatory mechanisms (A. P. Bird, 1995; A. P. Bird & Tweedie, 1995). Diversity emerges from the way genes are regulated during development as much as from genetic diversity itself, as made evident from the fact that all cells in an organism have the same complement of genes.

This information is not new but including development in our portrayal and in the empirical approach to complexity will help shape how we investigate the adaptive potential of complexity. Cells are not made of organs and individuals are not made of populations; organisms develop following a logical order and parts need components (Csete & Doyle, 2002; Valentine, 2003). Metazoans are not defined by a single level of hierarchical complexity and parts at each level are not randomly organized and assembled into parts at the next level (Carroll, 2001; McShea, 2000; McShea & Brandon, 2010; McShea & Changizi, 2003; Valentine, 2003). H. Simon (1962) and H. A. Simon (2002) characterized hierarchy as a crucial component of complexity, where hierarchical systems are defined as systems within which are found interacting subsystems which can be further decomposed into smaller subsystems themselves decomposable until the basic units of all subsystems are found. Early on, Simon emphasized hierarchy as an important quality of complex systems, a notion that is well acknowledged by modern definitions of complexity, more specifically in the concept of vertical complexity (Mcshea, 2017; McShea & Brandon, 2010).

Hierarchically nested levels are not isolated and will interact during early development and life history, and these interactions will guide phenotypic trajectories (Hallgrímsson, Lieberman, Liu, Ford‐Hutchinson, & Jirik, 2007). Parts at different levels of biological organization are linked by mechanical, molecular, electrical or other types of interactions that will influence their shape, their functionality and their topology (Csete & Doyle, 2002). However, these interactions and their sequence need to be inscribed in an overarching regulatory architecture to ensure that the proper interactions, both spatially and temporally, are achieved and that deleterious interactions are avoided. The more complex an organism is, the more interactions and feedback mechanisms will be required to ensure proper development.

Some early frameworks of complexity (Carroll, 2001; Simon, 1962) included interactions among parts throughout spatial or temporal scales as important properties of biological complexity. This approach acknowledged the idea that biological entities are not static and instead tend to change throughout time and in reaction to their environment. During a life cycle, interactions might be formed and dismantled, and these interactions play a key role in determining cell differentiation and their organization into tissues and organs. Cells communicate and integrate information from their interactions with other cells, tissues, and organs and from the environment as they grow, senesce, and are replaced. Depending on their developmental context, similar cell types can form different tissue types, or, conversely, different cell types might collaborate in the formation of a single tissue type (Figure 1). The dynamic nature of organisms, and the fact that all cells share the same genome, again emphasizes the need for a regulatory architecture overseeing the development and maintenance of complexity in organisms. While these frameworks did indeed acknowledge the dynamism of biological systems and the importance of developmental interactions, they tended to include interactions and levels of interactions as additional “layers” of complexity. Developmental interactions are pervasive through biological systems, not occurring on a single part or on a single level of biological organization but rather acting in a coordinated manner to produce a functional organism. Developmental interactions thus guide the assembly of biological complexity and treating them as additional “parts” might create confusion as to their relationship with biological complexity itself. For this reason, we argue for a conceptual separation between biological complexity and the interactions and level of interactions which constitute in part the regulatory architecture of biological complexity. We further argue that this regulatory architecture is found in the form of the “epigenotype” and “epigenetics” *sensu lato*.

### 1.2 Epigenetics

#### 1.2.1 Waddington and developmental epigenetics

Waddington defined the term “epigenetics” as the set of emergent properties and mechanisms that direct the development of organisms and integrate genotypic and environmental information to produce a functional individual (Jablonka & Lamm, 2011; Jamniczky et al., 2010; Waddington, 1942). The epigenotype as it was construed thus served as the “interpretative machinery” of the genotype, incorporating all the endogenous and exogenous sources of information thereby allowing gene expression to be fine‐tuned in response to environmental and developmental cues (Jablonka & Lamm, 2011; Klironomos, Berg, & Collins, 2013). Epigenetics *sensu* Waddington was epitomized by his metaphor of the epigenetic landscape (Jamniczky et al., 2010; West‐Eberhard, 2003, Figure 2a). In this visual metaphor, the development of a cell is represented as a rolling ball at the top of the landscape. The features of the landscape (i.e., the paths available for the ball) are determined by the genotype, including gene–gene interactions (epistasis) and pleiotropic effects as well as interactions between developmental processes, between developing cells and tissues among themselves and with the environment (West‐Eberhard, 2003). Waddington's epigenotype is thus the network of interactions underlying this landscape (West‐Eberhard, 2003). and epigenetic mechanisms are the causal interactions and mechanisms linking the genotype to the emergence of the phenotype (Jablonka & Lamb, 2005; Jamniczky et al., 2010).

**Figure 2:**
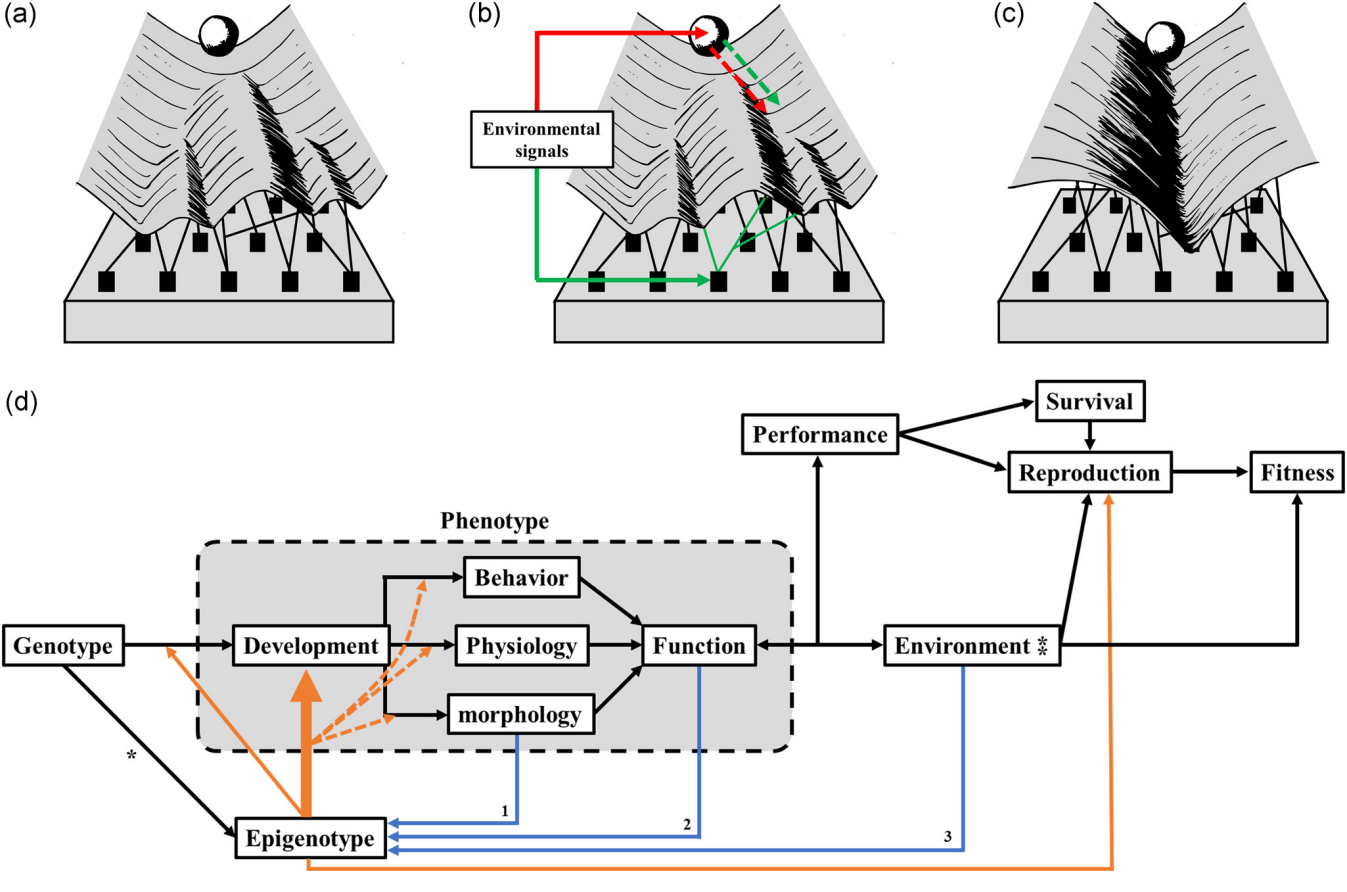
The epigenotype as the information‐processing machinery of the genotype. (a) The epigenetic landscape as imagined by Waddington. In this visual metaphor, cells start their development as rolling balls at the top of a slope. The features of the landscape then determine the available paths the ball can roll down (i.e., the different developmental pathways available to the cell). Waddington described the features of the landscape themselves being determined by the genotype: genes are drawn as pegs in a flat membrane, which are attached to the flexible landscape by strings representing molecular products, forming a network of gene products and gene interactions. These products can interface directly with the landscape or can interact with other products, forming a network of interaction affecting the landscape. (b) Within the epigenetic landscape, phenotypic plasticity can be represented in two ways. It can be visualized either as signals acting directly on the developing object (represented by the red arrows) or as signals affecting the interactions of the genetic network underneath the landscape, changing its topology and encouraging the ball to take a specific path (represented by the green arrows). (c) Canalization can be represented by a steep deepening of valleys within the epigenetic landscape, limiting the variation around the path the ball can take. (d) The epigenotype integrates endogenous and exogenous information to direct development. The genotype is in part responsible for the unfolding of developmental processes producing the phenotype, but information integrated by the epigenotype will affect the relationship between the genotype and development by directly regulating developmental processes involved in the genesis of behavior, physiology, and morphology. The phenotype of a given organism interacts functionally with its environment, determining its performance and expected fitness. The performance of an organism and its ability to survive and reproduce will shape its interactions with conspecifics and other organisms. The epigenotype is a network of interactions integrating information from different levels relating to the organism's phenotype, ecology, and its interaction with its ecosystem. The genotype determines what range of states the epigenotype can occupy. *The interaction between the genotype and the epigenotype includes both obligate epigenetics and facilitated epigenetics but not pure epigenetic variation which is either of the stochastic origin or of unknown environmental origin. (i) The morphology of an organism will provide feedback through physical interactions between tissues. (ii) The function will provide feedback information both by mechanical reception and tissue strain through use. (iii) The environment of an organism, including food sources, chemicals, and hormones in the environment, as well as abiotic parameters such as photoperiod, provides information on environmental quality and type which is integrated within the epigenotype. ⁑Environment includes biotic and abiotic characteristics of the external habitat as well as internal factors such as the microbiome of individuals and symbiotic organelles. Epigenetic mechanisms are also involved in reproduction, controlling how energetic resources are spent, either through behavioral mechanisms such as parental care or in biased energy allocation in eggs [Color figure can be viewed at wileyonlinelibrary.com]

Since Waddington formulated his concept of epigenetics, our understanding of the molecular and physical mechanisms responsible for gene expression and the development of the phenotype has changed dramatically (Jamniczky et al., 2010; Jablonka & Lamm, 2011). Epigenetic effects and mechanisms can be described as all effects and mechanisms not directly encoded by the DNA sequence, as well as emergent interactions between genes, and genes and gene products and could arguably include *cis*‐regulatory mechanisms (A. Bird, 2007; Hallgrímsson & Hall, 2011; Holliday, 2006; Jablonka & Lamb, 1998, 2005; Jamniczky et al., 2010). These include modifications to the structure of chromatin, gene silencing via RNAi, posttranscriptional modifications, methylation of DNA, and mechanical interactions between tissues among others (Jablonka & Lamb, 2005; Jamniczky et al., 2010; Johannes, Colot, & Jansen, 2008; O'Dea, Noble, Johnson, Hesselson, & Nakagawa, 2016). Epigenetic mechanisms are the basis of phenomena such as phenotypic plasticity and acclimation, as they allow for external stimuli to affect gene expression (Duncan, Gluckman, & Dearden, 2014; Jablonka & Lamb, 2005).

Waddington's original definition of epigenetics and the epigenotype was a very inclusive one, potentially grouping many mechanisms under a single unifying conceptual framework (Jamniczky et al., 2010). Today, however, epigenetic mechanisms and interactions have come to be classified under three separate categories: obligate, facilitated, and pure epigenetics (A. Bird, 2007; Gorelick, Laubichler, & Massicotte, 2011; Heard & Martienssen, 2014; Johannes et al., 2008; O'Dea et al., 2016; Richards, 2006, 2008). Obligate epigenetics are mechanisms and modifications that are proper to the genotype and remain uninfluenced by extrinsic factors (Johannes et al., 2008; O'Dea et al., 2016; Richards, 2008). They include epigenetic marks that are set during cell differentiation and participate in morphogenesis under normal circumstances (Johannes et al., 2008; O'Dea et al., 2016; Richards, 2008). Facilitated epigenetics include all effects and modifications which are dependent on genetic factors but influenced by environmental factors. Facilitated epigenetics are thus dependent on DNA sequences but may increase, or decrease, phenotypic variation expressed by their target sequences in response to environmental cues, or emergent interactions during development (Johannes et al., 2008; O'Dea et al., 2016). Pure epigenetics are those mechanisms where all variation is entirely independent of the DNA sequence and is due to environmental or stochastic effects only (Johannes et al., 2008; O'Dea et al., 2016). Stochastic developmental variation has been linked to adaptive strategies such as bet‐hedging (Vogt, 2015) and has been suggested to generate variation in adaptive traits such as tooth patterning in cyprinid fish and gill morphology and jumping behavior in mangrove *Rivulus* (Leung, Duclos, Grünbaum, Cloutier, & Angers, 2017; Turko, Earley, & Wright, 2011; Vogt, 2015).

#### 1.2.2 Role of epigenetics in evolution: plasticity and canalization

The epigenotype can integrate information from external sources and influence development to produce a cohesive organism that will be, to some extent, acclimate to its environment (Jaenisch & Bird, 2003; Figure 2a). For a given organism, while the genotype is static, the epigenotype can be modified during an organism's life and can incorporate changes in phenotypic interactions and the environment including community structure (Sobotka, Daley, Chandrasekaran, Rubin, & Thompson, 2016; Yan et al., 2014; Figure 2d). By incorporating environmental and developmental signals, different interactions might be created or dismantled during different stages of development or in response to environmental cues (Angers, Castonguay, & Massicotte, 2010; Gilbert & Epel, 2009). For example, community assemblages can trigger epigenetically mediated developmental processes (Yan et al., 2014). Sequential hermaphroditism in the genus *Amphiprion* is mediated by gender composition within a group (Iwata & Manbo, 2013; Iwata, Nagai, Hyoudou, & Sasaki, 2008). Groups of *Amphiprion ocellaris* are composed of a dominant female, a dominant male, and two or three subordinate males. The subordinate males are kept immature through aggression by the dominant male. When the female dies, the dominant male develops into a female and one of the subordinate males will grow to sexual maturity and replace the dominant male (Iwata & Manbo, 2013; Iwata et al., 2008). The presence of a female thus determines the phenotype of the dominant male, and the aggression from the dominant male delays the sexual maturing of the subordinate males (Iwata & Manbo, 2013; Iwata et al., 2008).

An important role of epigenetic mechanisms is to modulate the genotype‐phenotype relationship (Jaenisch & Bird, 2003; Bonduriansky & Day, 2009; Tronick & Hunter, 2016). Epigenetic mechanisms may facilitate the production of multiple phenotypes from a single genotype (Bonduriansky & Day, 2009; Costa, 2008; Tronick & Hunter, 2016), thereby producing diversity through plasticity. Phenotypic plasticity is the capacity for genotypes to produce multiple phenotypes in response to environmental stimuli (Pigliucci, Murren, & Schlichting, 2006, West‐Eberhard, 2003). The reaction norm, the pattern of phenotypic variation displayed over a range of environments, is dependent on how the interactions characterizing the epigenotype react to environmental cues. Interactions can be modified, created, or dismantled, leading to the development of environment‐specific phenotypes (Figure 2b; West‐Eberhard, 2003). Advantageous reaction norms, or parts thereof, can subsequently become fixed in the genome by genetic assimilation, the genetic encoding of effects that were induced by environmental factors (Jablonka & Lamb, 2005; Pigliucci et al., 2006; Waddington, 1961; West‐Eberhard, 2003). In the epigenetic landscape metaphor, plasticity can be conceived of in two distinct ways, both of which result in the ball being directed towards a specified valley. Environmental signals can be seen as acting on the ball itself, increasing the likelihood it falls within the specified valley, or they can act on the network of interactions underlying the landscape itself, effectively changing its shape so as to change the paths available to the ball.

Alternatively, and perhaps just as importantly, different genotypes can develop the same phenotype, or very similar phenotypes, a phenomenon known as canalization (Figure 2c; Debat & David, 2001; Hall, 1992; Hornstein & Shomron, 2006; Pál & Miklós, 1999). Canalization may prevent additional phenotypic variation from accumulating in a population despite genetic and environmental changes (Lande, 2009; O'Dea et al., 2016), either by limiting variation in gene expression levels or by exploiting genetic redundancy in pathways (Hornstein & Shomron, 2006). Canalization is visualized as a steep deepening of valleys in the epigenetic landscape, constraining the ball to a set path and reducing the variation around that path. Canalization may promote evolvability by allowing mutations to arise without affecting form and function, more mutations might accumulate and provide material for novelties to arise (Hornstein & Shomron, 2006; Lenski, Barrick, & Ofria, 2006; Masel & Trotter, 2010).

Epigenetic variation can also act as a dampener for genetic or environmental variation (Klironomos et al., 2013; O'Dea et al., 2016). Both canalization and plasticity can allow small populations or populations with low genetic diversity to survive bottlenecks and environmental instability (Klironomos et al., 2013; O'Dea et al., 2016). Plasticity can promote phenotypic diversity despite low genetic diversity and ensure that at least a fraction of the population might be positively selected, giving the population more time to evolve (S. M. Carlson, Cunningham, & Westley, 2014; Gibert, 2017; Lande, 2009). Alternatively, strong canalization might preserve a favorable phenotype in a population despite mutations and provide the population with more time to accumulate favorable mutations (de Visser et al., 2003; Lenski et al., 2006).

Epigenetic processes can thus direct phenotypic trajectories in response to environmental stress and selective pressures. Epigenetic mechanisms are involved in the generation of phenotypic variation in the absence of genetic variation as much as they are in limiting phenotypic variation in the face of genetic and environmental variation. The role that the epigenotype has in constraining or directing phenotypic trajectories is a context and trait‐dependent one, but the network of interactions depicted in Waddington's epigenotype underlie the many‐to‐many relationship between the genotype and the phenotype.

### 1.3 Interplay between epigenetics and complexity

#### 1.3.1 Epigenetics emerge from complexity in response to need for self‐regulation

While the link between epigenetics and development seems clear in the literature, investigations of the link among epigenetics, epigenetically mediated developmental processes and complexity remain sparse (but see Badyaev, 2014). This scarcity in the literature seems odd considering that the developmental role of epigenetic mechanisms, as first imagined by Waddington, where cells are the unit of action of epigenetic mechanisms, was to induce complexification and hierarchization of the phenotype (Hall, 1992, 1998; Waddington, 1942, 1957). We propose that the regulatory architecture of complexity is the “epigenotype” as defined by Waddington (1942): the network of interactions between cells during development, resulting in the shaping of tissues, structures, and organs (Jamniczky et al., 2010; Waddington, 1942, 1957).

**Figure 3:**
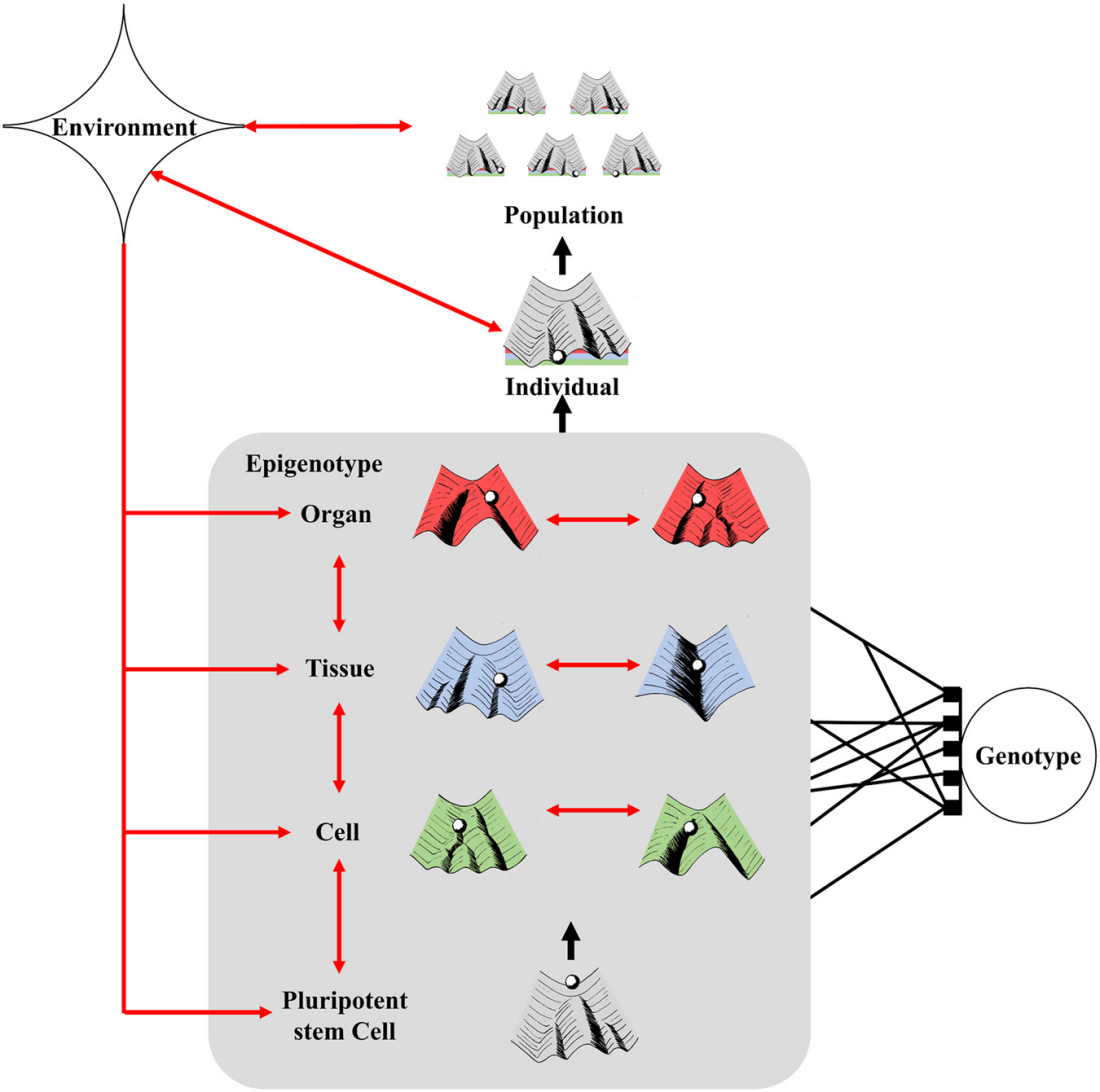
Vertical and horizontal complexity as a network of epigenetic interactions. The epigenotype includes interactions within systems and between systems across of nested levels of complexity defining an organism and incorporates environmental stimuli to direct the development of individual cells, tissues, or organs. At a given level of biological organization and time, complexity can be seen as an assemblage of epigenetic interactions shaping the development of individual parts (cells, tissues, organs, etc.), partly defined by the genotype of the organism. Each part at a given level of organization can be shown as having its own landscape which influences the topology of parts at higher hierarchical levels. The overlapping landscapes at each level are influenced by the genotype and merge to define the overall topology of the individual landscape, they constitute his epigenotype. The environment can influence individual and population dynamics, and individuals and populations can affect their environment. Environmental signals can affect the topology, or the paths taken by the ball in each landscape, at all scales of biological organization [Color figure can be viewed at wileyonlinelibrary.com]

We surmise that epigenetics is not only essential for the functional and viable development of complex organisms, but emerge from the spontaneous organization of increasing biological complexity during development (i.e., an increase in number of hierarchically nested levels of biological organization and an increase in number of parts and part types composing these levels throughout organismal development). This emergent set of properties, in turn, allows for biological complexity to be coordinated into a somewhat harmonious whole and serve as machinery to gather and interpret both endogenous and exogenous sources of information (Figures 2d and 3). This argument concords with Waddington's description of the epigenotype. Single‐cell organisms have limited interactions beyond the molecular level: they can interact with their environment which potentially includes other organisms. In more complex organisms, that is, multicellular organisms, interactions will be formed between the organisms and their environments, but interactions will also be formed within organisms between their constituent cells. When cells of multicellular organisms diversify, these cells will interact among themselves in new ways, and different cell types can interact with their environment in cell type‐dependent ways. As vertical complexity increases, new hierarchical levels of biological interactions will allow for new within‐level interactions to arise, as well as across‐level interactions. In addition, while hierarchically nested levels of complexity might be conceptually, and mathematically, independent from each other (there is no one‐to‐one correspondence between the number of parts across levels, e.g., multiple cell types can collaborate to produce a single type of organ or a single‐cell type can be assembled into many different organ types), they are not developmentally independent; systems or parts at one level are formed by parts at lower levels of biological organization. Communication between cell types becomes essential to ensure proper organ development and feedback participates in guiding the differentiation of new cells (Figure 3). As cell types and organs continue to diversify, interactions may steer from simple physical contacts to electrical signals (e.g., neurons) or molecular signals that can be diffused across bodies and manage distal interactions (e.g., hormonal secretion, neurotransmitters, and transcription factors). This network of interactions (Figure 3) thus emerges as both vertical and horizontal complexity increase, and while some of these interactions might fall outside of Waddingtonian epigenetics, most of them fit his definition.

#### 1.3.2 Modularity, integration, and costs of complexity

During development, parts of organisms, be they cells, tissues, or organs will interact and induce different phenotypic responses in each other. Yet, for the developing organism to be viable, and ecologically capable, certain structures need to be more resistant to variation in their surroundings and others might need to compensate for variation in surrounding structures. In other words, some structures need to be modular, whereas others need to be integrated. Modularity and integration can be described in terms of the variational properties of a system (Schlosser & Wagner, 2004; Wagner, Pavlicev, & Cheverud, 2007); modularity is the tendency of a system to vary independently of other systems. This means that the covariation between two independent modules should be virtually null (Mitteroecker & Bookstein, 2007; Wagner et al., 2007).

Both integration and modularity have genetic bases, but both can be altered epigenetically depending on endogenous and exogenous information. Constraints from mechanical sources or from functional demands tied to the ecology of an organism may be incorporated within their epigenotype (Jablonka & lamb, 2005; Maleszka, 2008; Newman & Müller, 2000). The importance of modularity is that it can constrain variation within modules but imposes few restrictions on other modules (Hulsey & Hollingsworth, 2011). The epigenotype further affects patterns of modularity by interpreting external signals from the environment. Conversely, morphological integration is the tendency for multiple traits or structures to vary in a coordinated fashion (Cheverud, 1982; Klingenberg, 2008; Olson & Miller, 1958; Wagner et al., 2007). Morphological integration plays a role in ensuring that function in complex morphological systems is not hindered by localized variation by inducing coordinated phenotypic responses within a module (Raff & Raff, 2000). Morphological integration is the result of physical interactions (for instance, mechanical interactions between tissues resulting from spatial packing Jamniczky et al., 2010), as well as genetic and epigenetic interactions (Gilbert & Epel, 2009; Jablonka & Lamb, 2002, 2005; Jamniczky et al., 2010; Zelditch, Wood, Bonett, & Swiderski, 2008). These mechanisms can create strong associations between tissues by regulating gene expression, modulating different developmental pathways either concomitantly or separately (Jablonka & Lamb, 2005; T. E. Parsons et al., 2011). Some amount of integration among parts in complex organisms is inevitable and results from the number of interactions characteristic of organismal complexity.

Connections between morphological integration and modularity with complexity are not new. The evolution of complexity potentially relies on some amount of integration within parts and some modularity among parts. Parts evolve into integrated systems because of strong selective pressures on their functionality (McShea, 2000), but for parts to evolve independently of one another, they need to be somewhat modular. Welch and Waxman (2003) proposed that modularity, or the dissociation between groups of genes or traits, could offset some of the evolutionary costs of complexity.

Modularity may mitigate costs by making individual modules more easily targetable by natural selection than if they were tightly integrated with other modules. Indeed, highly modular organisms are expected to be more evolvable than more integrated organisms (Villmoare, 2013) and modularity is expected to be favored when environmental conditions are unpredictable or for generalist ecological strategies (Clune, Mouret, & Lipson, 2013; Kane & Higham, 2015).

Morphological integration can also potentially promote complexity. Integration implies stronger correlations among traits, which intuitively might increase the costs associated with complexity in that traits lose their independence and a change in one might affect others (Wagner et al., 2007; Welch & Waxman, 2003). Integration results in a constraining of the degrees of freedom of variation (Klingenberg, 2008; Mitteroecker & Bookstein, 2007, Schlosser & Wagner, 2004). Even imperfect correlations among traits might reduce the variability of a system or constrain it enough to favor directional selection. However, integration can allow for structures interacting with a varying system to compensate for variation and maintain functionality. If a trait, or structure, is under strong directional selection, the variation of that trait might induce variation in surrounding traits to maintain morphological cohesion or compensate for new functional requirements (Klingenberg, 2008; Mitteroecker & Bookstein, 2007; Olson & Miller, 1958; Schlosser & Wagner, 2004).

#### 1.3.3 Change and stasis in complexity

Epigenetics and biological complexity seem inextricably linked and epigenetic mechanisms may participate either by maintaining current complexity, or by promoting changes in complexity in response to changing environments and selective pressures. Patterns of modularity are subject to change dependent on genetic and environmental contexts (Fischer‐Rousseau, Cloutier, & Zelditch, 2009; Zelditch, Lundrigan, & Garland, 2004); changes in the patterns of modularity and integration influence biological complexity by creating or breaking interactions between distinct systems or traits depending on selective pressures. Different patterns of modularity and integration will effectively change how traits or groups of traits respond to selective pressures.

As mentioned above, strong integration among traits can reduce the degrees of freedom of variation across groups of traits. Groups of traits would, therefore, be subject to selection in the same way as a single trait (Klingenberg, 2008; Mitteroecker & Bookstein, 2007; Schlosser & Wagner, 2004). Epigenetic interactions leading to strong integration among traits can also lead to reductions in complexity. The vertebrate cranium is an example of such a reduction in complexity (i.e., lower number of separable parts) promoted by increasing the number and strength of interactions among parts (Esteve‐Altava, 2017; Esteve‐Altava & Rasskin‐Gutman, 2015). This trend of reduction in complexity following increases in the number of interactions among parts at a given level may be expected in lineages undergoing functional specialization (Molnar, Esteve‐Altava, Rolian, & Diogo, 2017; Rueffler, Hermisson, & Wagner, 2012; Sidor, 2001), directing adaptive trajectories within these lineages.

The reverse can also be observed. For instance, the Weberian ossicles of ostariophysan fish mentioned earlier are derived from vertebral elements that initially evolved as serial homologs (N. C. Bird & Hernandez, 2009; Grande & de Pinna, 2004; Grande & Young, 2004). Neural and supraneural elements, as well as ribs, associated with the first four cervical vertebrae eventually became separated from corresponding centra and/or were modified into the Weberian ossicles and structural bones of the Weberian apparatus (N. C. Bird & Hernandez, 2007, 2009; Grande & de Pinna, 2004; Grande & Young, 2004). In this case, scission of elements, rather than fusion, accompanied functional specialization, despite numerous functional interactions among Weberian elements.

Similarly, epigenetic mechanisms might alter complexity in response to environmental signals. An example of a plastic reaction norm influencing complexity may be found in considering armor phenotypes in *Gasterosteus aculeatus* where the number and shapes of lateral plates changes in response to diet and parasitic load (Huang, 2015; Reimchen & Nosil, 2001). In this case, parts absent without an environmental cue could become expressed by the introduction of that cue. Complexity may also be stabilized by epigenetic mechanisms and need not necessarily be increased or decreased. Canalization could halt the appearance of new complexity and diversity when a given phenotype is favorably selected (McShea, 1991). Alternatively, phenotypes can change without the need for complexity to change, modularity can lead to topological changes in organisms, wherein individual structures remain largely unmodified but their position or organization within body plans will change. The importance of topology of organs and structures within body plans, despite being a crucial aspect of morphological disparity and variability, has only recently been reconsidered in evolutionary biology (Esteve‐Altava, 2017; Esteve‐Altava & Rasskin‐Gutman, 2015; Ronellenfitsch, Lasser, Daly, & Katifori, 2015). The morphological variation involving topological changes will not change complexity but may allow phenotypic variation for a fixed value of complexity within a lineage.

Decreases in complexity have been associated with terms like “catastrophe” or “drain”, which hold negative connotations (McShea, 2002; McShea & Brandon, 2010). However, decreases in complexity have occurred in multiple lineages. Evolutionary trends for decreasing complexity are usually the results of increased interactions within systems or strong integration and selection for streamlined functional traits (Csete & Doyle, 2002; McShea & Brandon, 2010; Wagner & Altenberg, 1996). Changes in complexity, whether increases or decreases, as well as stasis, have no intrinsic advantages but may be mediated by epigenetic processes to alter developmental strategies under different selective scenarios.

#### 1.3.4 Hybridization and hybrid complexes as models to study the link between epigenetic architecture and complexity

The evolution of complexity is an important topic in evolutionary biology, and its relationship to evolvability is clearly important. Understanding how the epigenotype and specific epigenetic mechanisms can participate in the evolution of both complexity and its relationship to evolvability are crucial to future inquiry. However, the study of epigenetics and their role in complexity is complicated by the fact that complexity changes and trends are difficult to track at the microevolutionary scale. Though complexity does vary across taxa within a genus or within families, major events of complexification are mostly discerned via morphological studies using the fossil record (Erwin, 2006; Valentine, Erwin, & Jablonski, 1996), a context in which the study of the interaction between epigenetic processes and complexity is significantly harder.

Complexity has been studied in laboratory animals, but laboratory‐reared models are often far removed from the biology of natural organisms and changes in complexity, either genetically or experimentally induced, often lead to nonfunctional or lethal phenotypes (Ehling, 1965; Suzuki et al., 2012). In addition, the absence in laboratory models of processes occurring in the wild, whose effects are essential to our understanding of evolution, makes some processes harder to understand in the context of environmental pressures (Jenner, 2006; Minelli & Baedke, 2014). Indeed, canalization is reduced in laboratory mice when compared to wild‐type mice and other murine lineages (Jamniczky & Hallgrímsson, 2009). Nevertheless, some natural organisms do provide interesting models of research to study changes in complexity and epigenetic architecture.

For instance, the gynogenetic hybrids from the *Chrosomus eos‐neogaeus* hybridization complex offer convenient natural models as they allow the study of genetic replicates in different environments (Castonguay & Angers, 2012). Meristic differences in the numbers of pharyngeal teeth and the presence or absence of a tooth row between *C. eos‐neogaeus* hybrid lineages seem to be linked to alternative developmental pathways triggered by environmental factors (Leung et al., 2017). In addition to meristic variation, pharyngeal arches with missing teeth presented alternative shape phenotypes to their complete counterparts (Leung et al., 2017). This change in shape could be a plastic response of the pharyngeal arch compensating for the loss of a tooth or tooth row (Leung et al., 2017). Here, both variation in the presence of teeth and teeth rows and divergence between paired symmetrical elements can be seen as changes in complexity. Individual hybrid genotypes are also known to display DNA methylation patterns that are strongly correlated to environmental differences, displaying increased environmental sensitivity and qualitatively different reaction norms compared to parents (Leung, Breton, & Angers, 2016); variation in these patterns of DNA methylation have been proposed as the mechanism responsible for the changes in teeth patterning and shape variation of pharyngeal arches within and between different genotypes of *C. eos‐neogaeus* (Leung et al., 2017).

Changes in epigenetic regulation within or across taxa, or as a result of disruptive processes such as hybridization can yield valuable information on how complexity changes when genomic coadaptation is disrupted and when new epistatic interactions are created quickly. Hybridization, the successful crossing between two distinct evolutionary lineages, has been known to disrupt conserved patterns of covariation and create phenotypic variation not present in parental species (i.e., phenotypic transgression), which can lead to changes in complexity (Barton, 2001; Dittrich‐Reed & Fitzpatrick, 2013; Renaud, Alibert, & Auffray, 2009, 2012). In some cases, hybrids have been known to display distinct patterns of integration and modularity from their parental lineages (Renaud et al., 2009, 2012). Hybridization is thus a process by which the evolutionary potential and the evolvability of hybrid, or introgressed, lineages, could be changed (Barton, 2001; Grant & Grant, 1994).

Hybridization can lead to drastic changes in complexity. Indeed, hybrids originating from interspecific crosses can display drastic differences with parental lineages, such as in the number and topology of skeletal elements in fish (Mills, Greenwood, & Peichel, 2014; Ou et al., 2018) or cytoplasmic elements (Deremiens, Schwartz, Angers, Glémet, & Angers, 2015). In the case of cytoplasmic hybridization, hybrids retain the nuclear genome from a parental species but incorporate cytoplasmic elements such as chloroplasts or mitochondria from the other species, effectively changing how the nuclear genome communicates with nonnuclear genomes and overall gene regulation (Deremiens et al., 2015). Changes in the numbers and number of types of organelles are a change in biological complexity affecting hybrids with important repercussions on their gene regulation and physiology.

Hybrid organisms are also known to display transgressive and novel phenotypes when compared to parental lineages. Some of this novelty is seen in second‐generation hybrids (F2), and later generations, as a product of genetic recombination and transgressive segregation, the production of new combinations of alleles in hybrids which were absent in parental populations due to genetic recombination (Kagawa & Takimoto, 2018; Rieseberg, Archer, & Wayne, 1999). New combinations of alleles may not be a change in complexity as such, although in the case of interspecific hybridization with parental lineages with genomes of diverging sizes it may be. However, these new combinations may lead to the expression of transgressive phenotypes which in turn may or may not involve changes in complexity. Conversely, first‐generation hybrids (F1), in which genetic recombination has not taken place, and which are generally expected to be phenotypic intermediates (Grant & Grant, 1994), may also display phenotypic novelties as a result not only of the combination of haplomes from different populations or species but also because of new epistatic interactions affecting gene expression and development (Galupa & Heard, 2015; Liu et al., 2017).

The production of phenotypic novelty can be exacerbated by changes in genomic complexity common in hybridization events such as ploidy elevations (Alix, Gérard, Schwarzacher, & Heslop‐Harrison, 2017; Freeling & Thomas, 2006), hybridogenetic hybrids (Dittrich‐Reed & Fitzpatrick, 2013) or rare cases of kleptogenesis such as seen in *Ambystoma,* where tetraploid populations of hybrids have been found with genomes containing up to three different genomotypes (Beauregard & Angers, 2018; Noël, Labonté, & Lapointe, 2011).

Hybridization was described as a mechanism capable of generating new lines of evolution (Grant & Grant, 1994). Indeed, the variety of effects hybridization may have on hybridizing lineages as well as on novel hybrid lineages are of interest to evolutionary biologists and the role of ongoing or ancient hybridization in the evolutionary history of extant taxa has generated a lot of research (Albertson & Kocher, 2005; Meier et al., 2017; K. J. Parsons, Son, & Albertson, 2011, Payseur & Rieseberg, 2016; and see Abbott et al., 2013 and Soltis, 2013 for comprehensive reviews). Hybrids are potent models for the study of complexity because of the potential for transgressive and novel, and sometimes extreme, phenotypes absent in parental lineages. While hybridization is not the only mechanism by which novelties may appear, novel phenotypes arising from hybridization occur within a few, or sometimes a single generation. The role of epigenetic mechanisms in the maintenance and regulation of complexity in hybridization is a topic that has garnered very little attention. Yet, hybridization zones and complexes might yield crucial new information on how changes in epigenetic architecture allow for quick changes in complexity to be functionally organized and how epigenetically mediated modularity or developmental decoupling of traits can alter evolutionary trajectories.

## 2 CONCLUSION

The evolution of biological complexity has been a central focus of systems biology, yet complexity has only seldom been addressed directly in the evo–devo literature despite its relevance to the study of developmental interactions and constraints. The study of complexity has been hindered by the lack of consensus on its definition, but recent work has reduced the definitions of complexity to its purest and simplest dimensions: the number of different parts at each hierarchical level of biological organization and the number of hierarchically nested steps defining the object of study. Although “complexity” defined this way makes the term operational, the modification and assembly of parts in biological systems requires a regulatory control of some sort which likely rests in the form of the epigenotype *sensu* Waddington.

Clear and separate definitions for complexity and epigenetics are important for their individual understanding and to investigate relationships between the two, however, complexity has seldom been investigated in light of epigenetics to the point of empirical studies treating complexity as insulated from any sort of biological constraint or group of mechanisms. We believe this empirical separation between biological complexity and biological constraints and mechanisms could lead to unfortunate overlooks, impeding the study of complexity and hope the present paper will provide a starting point for research bridging complexity to developmental constraints and mechanisms.

We have argued that the epigenotype, and epigenetic mechanisms, are not only the regulatory architecture for complexity but potentially emergent properties of complexity itself. Epigenetically mediated morphological integration can act as an important process in the evolution of complexity and in the optimization of organismal complexity to tailor phenotypic reaction norms to selective and environmental pressures. Indeed, modularity and integration can distribute selective forces across traits or keep them limited within a structure, affecting the reactions norm of organisms without necessarily sacrificing complexity.

These ideas, though they do not necessarily explain increases or decreases of biological complexity during evolution on their own, should be incorporated within our conceptions of biological complexity and its evolution. They open new questions and avenues of research and contribute to old lines of inquiry that still have not found answers, for instance: does biological complexity have an upper limit? Selection on the basis of performance and integration in functional systems seem to suggest that complex systems might become streamlined and that complexity will eventually be reduced. In addition, increasing the number of interactions among systems seems to lead to complexity reductions such that there might be an upper limit as to how many parts can interact without incurring subsequent fusion of parts or decreases in their numbers. Considering recent research on nongenetic inheritance, the effects of intergenerational epigenetic inheritance on complexity could also be explored. Finally, while integration and modularity do shape developmental and evolutionary trajectories, the degree to which changes in morphological integration can constrain or release the appearance of complexity and the mathematical relationship between the two remain unanswered and important questions.

## CONFLICT OF INTERESTS

The authors declare that there are no conflict of interests.
